# The Familial Intracranial Aneurysm (FIA) study protocol

**DOI:** 10.1186/1471-2350-6-17

**Published:** 2005-04-26

**Authors:** Joseph P Broderick, Laura R Sauerbeck, Tatiana Foroud, John Huston, Nathan Pankratz, Irene Meissner, Robert D Brown

**Affiliations:** 1 Department of Neurology, University of Cincinnati, 231 Albert Sabin Way, Cincinnati, OH 45267-0525, USA; 2 Medical & Molecular Genetics, Indiana University, 975 West Walnut St., IB 130, Indianapolis, IN 46202-5251, USA; 3 Department of Radiology, Mayo Clinic, 200 First Street SW, Rochester, MN 55905, USA; 4 Division of Cerebrovascular Disease and Department of Neurology, Mayo Clinic, 200, First Street SW, Rochester, MN 55905, USA

## Abstract

**Background:**

Subarachnoid hemorrhage (SAH) due to ruptured intracranial aneurysms (IAs) occurs in about 20,000 people per year in the U.S. annually and nearly half of the affected persons are dead within the first 30 days. Survivors of ruptured IAs are often left with substantial disability. Thus, primary prevention of aneurysm formation and rupture is of paramount importance. Prior studies indicate that genetic factors are important in the formation and rupture of IAs. The long-term goal of the Familial Intracranial Aneurysm (FIA) Study is to identify genes that underlie the development and rupture of intracranial aneurysms (IA).

**Methods/Design:**

The FIA Study includes 26 clinical centers which have extensive experience in the clinical management and imaging of intracerebral aneurysms. 475 families with affected sib pairs or with multiple affected relatives will be enrolled through retrospective and prospective screening of potential subjects with an IA. After giving informed consent, the proband or their spokesperson invites other family members to participate. Each participant is interviewed using a standardized questionnaire which covers medical history, social history and demographic information. In addition blood is drawn from each participant for DNA isolation and immortalization of lymphocytes. High- risk family members without a previously diagnosed IA undergo magnetic resonance angiography (MRA) to identify asymptomatic unruptured aneurysms. A 10 cM genome screen will be performed to identify FIA susceptibility loci. Due to the significant mortality of affected individuals, novel approaches are employed to reconstruct the genotype of critical deceased individuals. These include the intensive recruitment of the spouse and children of deceased, affected individuals.

**Discussion:**

A successful, adequately-powered genetic linkage study of IA is challenging given the very high, early mortality of ruptured IA. Design features in the FIA Study that address this challenge include recruitment at a large number of highly active clinical centers, comprehensive screening and recruitment techniques, non-invasive vascular imaging of high-risk subjects, genome reconstruction of dead affected individuals using marker data from closely related family members, and inclusion of environmental covariates in the statistical analysis.

## Background

Stroke is the third leading cause of death and the leading cause of disability among adults. Subarachnoid hemorrhage (SAH) due to rupture of IAs is one of the three main subtypes of stroke. The incidence of SAH as well as its 30-day mortality has remained stable for over 3 decades despite advances in diagnosis and treatment [1-4]. Survivors are often left with substantial disability and a reduction of quality of life [5,6]. Most of the mortality after rupture of an IA is due to rapid and massive brain injury from the initial bleeding that is not correctable by medial and surgical intervention [7]. In addition, unruptured IAs are estimated to be present in at least 1.0% of the general population [4]. Unruptured IAs are associated with a variable risk of aneurysmal rupture that increases with size of the aneurysms [8,9]. Thus, the most effective ways to decrease morbidity and mortality associated with IAs are to prevent formation of IA in the population and to identify asymptomatic unruptured IA in affected individuals prior to rupture.

Non-modifiable risk factors for aneurysmal SAH include advancing age, female gender and African American race [2,3,10-12]. Smoking and hypertension are the modifiable environmental risk factors that have been very strongly linked to intracranial aneurysms and SAH whereas heavy alcohol use and other environmental factors have been less consistently linked [13-19].

Population-based and case-control studies suggest that genetic factors also play an important role in the formation and rupture of IA [18,20-22]. For example, the risk of unruptured IA, as determined by magnetic resonance angiography (MRA) screening of unaffected relatives in families with two or more members who have an IA, is about four times greater than the risk among the general population [23]. Several Mendelian disorders, such as polycystic kidney disease and Ehler's Danlos syndrome (particularly Type IV), are associated with an increased risk of IA formation. However, these disorders account for less than 1% of all IAs in the population and therefore cannot explain the familial aggregation of IA [10].

Several studies have sought to identify the genes contributing to IA susceptibility. Analyses of potential candidate genes for IA, such as α-1 antitrypsin, apolipoprotein E, and lipoprotein lipase, have yielded inconsistent results and appear unlikely to explain the substantial genetic risk for IA [24-30]. Linkage of FIA in two relatively small linkage studies to a region on chromosome seven containing the elastin gene has not been confirmed by other studies [31,32]. Another small linkage study in a Finnish population has identified a linked region on chromosome 19 that has yet to be confirmed by another study [33]. Most recently, a single, large family segregating an apparently autosomal dominant form of IA has been linked to chromosome 1p [34]. Thus, the genetic cause of IA remains largely unknown. The long-term aim of the Familial Intracranial Aneurysm Study is to identify genes that increase the risk of development and rupture of IAs.

A critical limitation in a number of family studies of IA is the significant early mortality. As a result, collection of a sufficient number of families with affected individuals can severely limit the success of potential genetic studies. One approach to improve the power to detect IA susceptibility genes is to recruit families with multiple members with IA, and to enroll the children and spouses of deceased affected in these families. Similar to the approach used in forensic studies, analysis of the DNA of closely related individuals of the deceased person allows for the reconstruction of the likely genotype of the deceased, affected person- thereby improving the power of genetic analyses. A second approach to improve the power of IA studies is to perform MRA studies among high-risk family members so as to identify individuals with unruptured aneurysms. When performing genetic analyses, those individuals with unruptured IA will be considered affected and thus improve the power of genetic analyses. A third approach to further increase the power of genetic studies is to perform analyses to detect loci contributing to IA susceptibility and/or rupture risk. In this way, unique and potentially overlapping loci contributing to each effect can be identified.

## Methods and Research Design

### The FIA collaborative group

The FIA Study consists of the Coordinating Center (University of Cincinnati), two genotyping centers (The Center for Inherited Disease Research and University of Cincinnati), the Imaging Center (Mayo), the Cell Repository (Coriell), two Statistical Genetics Centers (Indiana University and National Human Genome Research Institute) and 26 clinical centers. To maximize the ability to recruit families with IA, clinical centers with extensive experience in the clinical management and imaging of intracerebral aneurysms were selected as recruitment sites. These centers (41 recruitment sites) are located throughout North America, New Zealand, and Australia. The FIA study has been approved by the Institutional Review Boards/Ethics Committees at each of the study centers, recruitment sites and participating Centers.

### Definition of phenotype

The primary phenotype is an intracranial aneurysm which is a berry-like defect in the wall of an intracranial artery at the base of the brain. Individuals with both ruptured and unruptured IAs are considered to have the phenotype.

### Study population

Four hundred seventy-five families with multiple members diagnosed with IA will be enrolled to identify the chromosomal regions associated with an increased risk of IA and to determine the effects of environmental factors on the expression of genes within these regions. Eligible families for this study include: 1) Families with at least 2 living affected siblings. 2) Families with at least 2 affected siblings, one of whom is living and the other whose genotype can be reconstructed through the collection of closely related, living family members [i.e. Spouse and children] 3) Families with ≥ 3 affected family members (e.g. cousin, uncle, aunt), two of whom are alive and have living connecting relatives. 4) Families with ≥ 3 affected family members, with one living affected and at least one other affected relative whose genotype can be reconstructed through the collection of closely related, living family members.

Exclusion criteria include a fusiform-shaped unruptured IA of an intracranial artery; an IA which is part of an arteriovenous malformation; a family history of polycystic kidney disease, Ehlers Danlos Syndrome, Marfan's Syndrome, fibromuscular dysplasia or Moya-Moya syndrome; or failure to obtain informed consent from the patient or family members.

### Probands

Probands are the first living person identified within a family, who have had a confirmed diagnosis of an IA and do not meet any of the exclusion criteria.

### Other affected family members

The proband, or family members of the proband, contacts other potentially affected family members to determine their willingness to participate in the study. If they are agreeable, a study coordinator makes contact with the additional affected family members. For deceased and living relatives with a family history of IA, SAH or intracerebral hemorrhage (ICH), medical records (e.g. hospital records, imaging reports, autopsy, death certificate) are requested after the patient's or family's permission has been obtained. In addition, a phone screen is completed to document the symptoms, diagnostic testing, and management of a diagnosed IA.

### Verification of phenotype

All medical records and the phone screen of probands and family members with a reported history of IA, SAH or ICH are reviewed by a Verification Committee. This committee consists of study neurologists at the University of Cincinnati and The Mayo Clinic. Two neurologists independently review the records and decide if the subject meets all the inclusion and exclusion criteria. In cases of disagreement, a third neurologist is used to resolve the case diagnosis as a tiebreaker. Each potential affected family member is ranked as:

#### Definite

Medical records document aneurysm on angiogram, operative report, autopsy, or a non-invasive imaging report (MRA, CTA) demonstrates an IA measuring greater than 7 mm.

#### Probable

Death certificate mentions probable intracranial aneurysm without supporting documentation or autopsy. Death certificate mentions subarachnoid hemorrhage without mention of aneurysm and a phone screen is consistent with ruptured IA (severe headache or altered level of consciousness [LOC]) rapidly leading to death. An MRA documents an IA that is less than 7 mm but greater than 3 mm.

#### Possible

Non-invasive imaging report documents an aneurysm measuring between 2 and 3 mm. Subarachnoid hemorrhage was noted on death certificate, without any supporting documentation, autopsy or recording of headache or altered LOC on phone screen. Death certificate lists 'aneurysm' without specifying cerebral location or accompanying SAH.

#### Not an affected Case

there is no supporting information for a possible IA.

### Enrollment of non-affected family members

Once the eligibility of the family is established, all first degree relatives of the affected family members who have expressed interest in the study are contacted and enrolled. If the affected person is alive, the first-degree relatives, including the brothers and sisters of the affected person, are contacted for enrollment. If the affected person is deceased, then the brothers and sisters as well as the spouse and children of the affected person are recruited for the study. These additional family members, particularly the spouse and children, will be used to reconstruct the genotype of the deceased, affected individual. To further improve the power of genetic analyses, when the family consists of pairs of affected family members who are more distantly related (i.e. cousins, avuncular, etc), linking relatives such as aunts, uncles and parents are enrolled into the study.

### Recruitment methodology

Every new case of IA or SAH is screened for a family history of IA or intracranial hemorrhage at the study clinical centers. To ensure adequate enrollment, each selected clinical site is a major referral center for the diagnosis and treatment of IAs. We utilize three recruitment strategies that include prospective monitoring of all IA cases at the clinical centers, retrospective review of IA cases, and advertisement on the internet and in scientific journals. To help with accessibility to the coordinating center, a study web site  and toll free number (800-502-4-3427) has been established.

An unexpected source of potential FIA families has been direct contact of the FIA study personnel by potential probands who have heard about the study through print, radio or TV news media. This source of referrals has resulted in the identification of 77 (19%) of the first 405 families. Use of all four recruitment methods has lead to a four-fold increase of identified potential families over the expected rate of recruitment. At the end of the first 19 months, 405 families have been identified, as compared to an expected 164 families.

### Enrollment of study subjects

Once a potential family is identified and the permission of the treating physician has been obtained, the proband or their proxy is given an information sheet describing the study and is asked to enroll into the study. Prior to giving informed consent, a subject must pass the short version of the Blessed Information-Memory-Concentration Scale (Short Blessed Test). This scale assesses the presence and severity of significant cognitive impairment. All six items are applicable to subjects whether in the home or in an institution, can be administered in person or over the phone, and can be applied to persons with physical handicaps (e.g. blindness). In accordance with local regulations, if the subject does not pass the Short Blessed Test, a proxy is chosen then a medical history questionnaire is administered and a blood sample is taken. A phone screen is completed and release of medical information is obtained for phenotype verification. The proband or their proxy representative is then asked to complete a family history questionnaire; study information sheets and recruitment letters are provided to be distributed to additional family members.

Interested family members who live at a distance from a study site are contacted by the Study Coordinating Center and informed consent is obtained via phone, mail or fax. The medical history questionnaire is obtained via phone. A home health agency, which has contracted with the study, then collects the blood samples and obtains the blood pressure readings.

### MRA screening of FIA family members without known IA

MRA, a non-invasive imaging technique of cerebral vessels that images the movement of protons, has been demonstrated to be an effective method to screen for IA in asymptomatic relatives. Compared to the gold standard, cerebral angiography, the sensitivity of MRA in detecting IA ranges from 81 to 95 % (most studies greater than 90%) [35-43]. The interobserver consistency in identifying IA's by MRA, particularly for IA's >3 mm is good to excellent (kappa 0.59–0.82) [35-41,43].

Identification of asymptomatic IA cases by MRA provides additional living affected individuals to be used in linkage analysis. To maximize the ascertainment of asymptomatic IAs and to minimize the costs of imaging associated with the study, a study protocol is used to identify individuals at high risk for IA. This protocol calls for the imaging of first degree relatives of affected subjects who are 30 years of age or greater, have a history of hypertension or a mean blood pressure of > 140 mmHg systolic, or >90 mmHg diastolic and/or a 10 pack-year history of smoking.

Patients referred for MRA are imaged at a limited number of certified sites. To obtain certification, the site must demonstrate the ability to perform high quality MRA according to certain standards identified by the Imaging Center at the Mayo Clinic. All study MRAs are de-identified prior to shipment to the Imaging Center where they are reviewed by two neuroradiologists. Agreement between the 2 readers is declared when both detect no aneurysm or both identify an aneurysm at the same site with a measurement difference of no greater than 1 mm. When a disagreement between the readings of the two neuroradiologists occurs, a consensus reading is complete. If consensus cannot be reached, adjudication by a third, blinded reader is performed.

When an IA is identified by MRA, the Coordinating Center notifies the Principal Investigator (PI) at the site of subject enrollment. The local PI contacts the subject, advising them and their physician of the findings. Clinical management of those with unruptured IAs is not dictated by the study.

### Yearly follow-up

Once a year, a medical information update and quality of life form is obtained from each participant. This form provides a means of maintaining contact with participants as well as collecting information concerning any deaths or new cases of IA that may have occurred since completion of the Family History Questionnaire.

### DNA collection, abstraction, and initial genotyping

Each FIA site is provided with blood collection kits. Two tubes collected in EDTA are used by DNA extraction and a third tube is used for isolation and immortalization of lymphocytes at Coriell, the DNA repository for the National Institute of Neurologic Disorders and Stroke. The extracted DNA is batched and sent to The Center for Inherited Disease Research (CIDR). When it has been received by CIDR a 10 cM genome scan is performed using automated fluorescent microsatellite analysis

### Reconstruction of genotypes

To improve the power of genetic analyses despite the high mortality of this condition, reconstruction methods are used to infer the genotypic data of a missing individual. Prior to initiating the study, three-generational pedigrees were simulated consisting of a pair of siblings, their deceased parents, one of the sibling's spouses, and three offspring. A marker with heterozygosity of 70% was simulated. Then, the genotypic data of the sibling with the spouse and offspring were removed. Analyses were then performed to estimate the accuracy with which the missing individual's genotype could be inferred using a sequentially smaller number of relatives. Six conditions were tested: a) three offspring, with and without DNA of the deceased individual's spouse; b) two offspring, with and without the deceased individual's spouse; and c) one offspring, with and without the DNA of the deceased individual's spouse.

The program Allegro was used to infer the genotype of the missing individual [44]. The haplotyping algorithm in Allegro was used to impute the most likely genotype of the missing individuals. The inferred genotype was tabulated for the missing individual and the percentage of families in which the correct genotype was inferred was determined. Results were independently obtained for each of the six family structures with varying numbers of individuals available for assisting in the reconstruction of the missing individual. The proportion of families in which the genotype was correctly inferred was clearly dependent on the number of family members available for genotyping. When three offspring and a spouse were available, the correct genotype was inferred as the most likely genotype in about 90% of the families. Even in the worst-case situation, in which only 1 child was available to assist in the reconstruction, the correct genotype was inferred in 73% of the pedigrees. These data demonstrate that the collection of family members of a critical, deceased individual is an effective way to infer the genotype of a missing affected subject.

### Statistical methods

Our proposed collection of families will initially consist of families with at least one sib pair (3/4 of families) and families with three or more affected persons but no sib pair (1/4 of families) prior to MRA screening. However, through MRA screening to identify additional asymptomatic individuals, we anticipate that 1/3 of our sib pair families will now consist of a third affected individual. In addition, 1/3 of the other 100 families which initially did not include a sibling pair but had 3 or more affected relatives, will now also include a sibling pair.

To identify chromosomal regions linked to IA, a genome screen will be performed using polymorphic markers genotyped at regular intervals across the genome. Two complementary approaches will be initially employed to detect chromosomal regions linked to the risk for IA. First, parametric methods (i.e. Allegro) will be employed, with the risk for IA modeled as a reduced penetrance, autosomal dominant disorder. A conservative approach to the analysis will be implemented using only the affected individuals; unaffected individuals will be considered as having unknown phenotype. Second, nonparametric method (i.e. Merlin) will also be used to detect linkage. Allele sharing will only be estimated using affected individuals. In both the parametric and nonparametric analytic methods, the genotypes of the unaffected individual are used to infer the genotype of deceased, affected individuals. Both analytic approaches will be performed with both a narrow and broad disease definition. Under the narrow disease definition, individuals will be considered affected only if they are classified as having a definite aneurysm. The broader disease definition will include as affected those individuals with either definite or probable aneurysm.

Since the genotyping of the familial sample will be performed in stages, the initial goal of all linkage analyses is to detect chromosomal regions providing evidence of linkage to IA. Since these studies are an initial screen, modest genome-wide criteria for linkage will be employed. By simulating genotypes for the sampled pedigrees, genome-wide thresholds for linkage can be determined. Initial criteria will employ the 5% and 1% threshold for linkage.

Once linkage has been identified, further analyses will be performed to dissect the likely mechanism of action. Initially, the genetic analyses will be performed using as affected only those individuals whose IA had ruptured. This analysis is designed to determine if the linkage that has been detected is to a locus contributing to IA or to the risk of IA rupture. Subsequent analyses designed to further localize the chromosomal region linked to IA will utilize more effectively the 'unaffected' individuals. Specifically, data from important environmental covariates such as smoking and hypertension will be used to modify the risk an individual has inherited an IA susceptibility gene.

There are few analytic methods currently available that can perform genetic linkage analysis while simultaneously considering the risk of various non-genetic risk factors. Conditional logistic models that include covariates within the model allow the genetic relative risk to depend on the covariate [45-47]. Recent application of this technique suggest substantial improvement in the ability to detect apparently 'true' linkage signals across multiple datasets, which were undetected using conventional linkage analyses [46]. Another approach is a regression-based extension of the mixture likelihood, which includes pedigree features as covariates to determine the probability that a pedigree is linked [48]. For example, this approach was applied to prostate cancer and examined the family effects of the number of affected individuals in the pedigree, mean age at diagnosis and male-to-male transmission [47]. These investigators found that the covariate of male-to-male transmission identified a subset of families with evidence of a unique linkage. Importantly, they, as well as others, found that including insignificant covariates in the linkage analysis reduced the power to detect linked chromosomal regions [49]. A new approach recently applied to sibling pair data is a tree-based recursive partitioning method that produces more homogeneous subgroups of sibling pairs, which can lead to substantial increases in the power to detect linkage [48].

## Discussion

A successful, adequately-powered genetic linkage study of IA is challenging given the very high early mortality of ruptured IA. Other diseases such as lung cancer also have a high associated mortality but patients survive months and even years after diagnosis whereas 40% of patients with a ruptured IA die within the first month. This clinical reality necessitates very aggressive recruitment of potential probands who have a ruptured IA within the first week or so after admission to the hospital. This aggressive approach to prospective recruitment of cases, retrospective case identification at very active clinical centers, and extensive coverage of the study in the media has greatly enhanced our recruitment of families over initial expectations.

Accurate and reproducible phenotyping is the key to any genetic linkage study. IA, in general, is a much more clearly defined phenotype than other neurologic diseases such as Parkinson's disease, Alzheimer's Disease, or even ischemic stroke where the signs and symptoms may be very subtle and poorly documented. IA is defined by a very clearly observed anatomic vascular defect visible on vascular imaging, at operation, or at autopsy. In addition, the large majority of subarachnoid hemorrhage is due to rupture of IA. The challenge of phenotyping for IA is the rapid mortality which precludes vascular imaging and may also result in incomplete information in the available medical records or death certificates. For example, a death certificate may be the only medical record and it may clearly indicate that a massive suabarachnoid hemorrhage was the cause of death, but does not mention an IA. To address these issues, we have designed a rigorous phenotyping procedure using medical records, family interviews, and death certificates with carefully defined levels of confidence (definite, probable, and possible). Thus, we will perform a primary statistical analysis using only those individuals who meet the criteria for definite or probable and a secondary analysis that also includes the possible cases.

Phenotyping is tied closely to imaging of cerebral arteries. Intra-arterial angiography, the gold-standard for identification of IAs, has an associated risk of stroke of 0.5–1.0% [50-55]. Major advances in non-invasive imaging of cerebral arteries over the past 10 years have dramatically changed the approach to IAs and have resulted in identification of additional affected individuals in genetic linkage studies of IA with an increase in study power [31,33,56]. However, inclusion of individuals as affected based upon MRA imaging alone, without confirmation by intra-arterial angiography, runs the potential risk of inclusion of some subjects in the analysis as "affected" who do not actually have an IA. Many physicians do not advocate invasive intra-arterial angiography to confirm IA detected by MRA or CT angiography unless there is serious consideration of clipping or coiling of the IA. Often, only those patients with aneurysms that are 5 mm or larger on MRA or CT angiography undergo subsequent intra-arterial angiography. To address this issue of phenotyping using MRA or CT angiography, we have required high-quality methods for MRA imaging at the clinical centers and have developed a rigorous protocol for centralized identification of MRA by highly experienced neuroradiologists. Our phenotyping of IA using MRA (definite, probable, and possible) is also defined by size of the IA since sensitivity and specificity of MRA for identification of IA clearly relates to the size of IA.

This study highlights several relatively novel approaches to increase the power of genetic analysis. The approach to genotype relatives of deceased, affected individuals is critical in genetic studies of IA as well as other high mortality disorders. Our modeling of various pedigree structures indicates that reconstruction of the most probable genotype of a dead affected can be determined by including the spouse and children. Another important aspect of IA genetic studies is the critical need to consider environmental covariates in disease risk. Specifically, inclusion of smoking as a risk factor for IA is essential since nearly 80% of patients with an IA have a history of smoking at some point during their lifetime and the attributable risk associated with smoking at any time for IA is over 50% [18]. However, not all individuals who smoke develop IA. Therefore, we hypothesize that smoking may substantially increase the risk of IA for individuals with particular genotypes at IA susceptibility loci. Therefore, to accurately model this interaction and localize the genes contributing to IA, it is critical to effectively incorporate key environmental covariates in genetic linkage and association analyses

In summary, the NINDS-funded FIA Study is by far the largest genetic linkage study of IA to date. The FIA Study has excellent power to detect genes associated with the development of rupture of IA and has a unique opportunity to understand the complex interrelationships between genes and environment that lead frequently to a deadly outcome.

## List of Abbreviations

CIDR, The Center for Inherited Disease Research

FIA, Familial Intracranial Aneurysm

IAs, intracranial aneurysms

ICH, intracerebral hemorrhage

LOC, level of consciousness

MRA, magnetic resonance angiography

NINDS, National Institute of Neurological Disorders and Stroke

PI, Principal Investigator

SAH, subarachnoid hemorrhage

## Competing Interests

The author(s) declare that they have no competing interests.

## Authors' Contributions

JPB conceived of the study and participated in its design and coordination and helped to draft the manuscript. LRS participated in the design and coordination of the study and drafted the manuscript. TF participated in the design of the study statistical methods and the reconstruction of genotypes and helped to draft the manuscript. JH participated in the design of the study imaging methods and helped to draft the manuscript. NP participated in the methods genotype reconstruction. IM participated in the design of the study. RDB participated in the design and coordination of the study and helped to draft the manuscript. All authors have read and approved the final manuscripts.

## Appendix

### Study Operational Centers

*Coordinating Center *– University of Cincinnati: J. Broderick, principal investigator ; D. Kleindorfer, co-principal investigator; L. Sauerbeck, study coordinator; S. Ewing, administrator; J. Sester, research assistant; *Genotyping Center *– University of Cincinnati: R. Deka, principal investigator; D. Smelser, research assistant;*Linkage Analysis *– Indiana University: T. Foroud, principal investigator; P. M. Conneally, co-principal investigator; M. Daubs, Data Manager; J. Gray, research coordinator; L. Flury, statistician; *Imaging Center *– *Mayo Clinic*: J. Huston III, co-principal investigator; D. Kallmes, study neuroradiologist, M. Maronie Smith, MRI study coordinator: *Cell Storage Center *– Camden, New Jersey: J. Beck, C. Royds,; National Institute of Neurological Disorders: J. Marler; K. Gwinn-Hardy.

### Recruitment Centers

*University of Alabama at Birmingham*: W. Fisher, principal investigator, H. Forson, coordinator; *Auckland New Zealand: *C. Anderson, principal investigator, E. Mee, co-principal investigator, C. Howe, coordinator, S. Vos, coordinator: *Australia*: G. Hankey, principal investigator, P. DUrso, principal investigator, N. Knuckey, principal investigator, J. Laidlaw, principal investigator, P. Reilly, principal investigator, N. Dorsch, co-principal investigator, M. Morgan, principal investigator, M. Besser, principal investigator, K. Athanasiadis, coordinator, Claxton, coordinator, J. Davidson, coordinator, V. Dunne, coordinator, S. Ewen, coordinator, J. Griffith, coordinator, S. Pope, coordinator, J. Raftesath, coordinator, E. Ritson, coordinator; *Brigham & Women's Hospital: *A. Day, principal investigator, J. O'Hare, coordinator; *University of Cincinnati*: D. Woo, co-principal investigator, M. Zuccarello, co-principal, A. Ringer, co-principal investigator, H. Yeh, co-principal investigator, K. Franklin, coordinator; *Cleveland Clinic Foundation*: P. Ramussen, principal investigator, D. Andrews-Hinders, coordinator; *Columbia University*: E. S. Connolly, principal investigator, R. Sacco, co-principal investigator, R. Ellsasser, coordinator, P. Yung, coordinator; *University of Florida*: S. B. Lewis, principal investigator, R. Dettorre, coordinator, A. Royster, coordinator; *Indianapolis Neurosurgical Group*: T. Payner, principal investigator, N. Miracle, coordinator, K. Redelman, coordinator; *London Health Science Center Research Inc*.: G. Ferguson, principal investigator, C. Mayer, coordinator, J. Peacock, coordinator; *John Hopkins University*: K. Murphy, principal investigator, B. Kohler, coordinator; *Massachusetts General Hospital: *C. Ogilvy, principal investigator, D. Buckley, coordinator, T. Taytsel, coordinator;*McGill University*: G. Rouleau, principal investigator, A. Noreau, coordinator, N. Satge, coordinator; *University of Maryland*: E. F. Aldrich, principal investigator, C. Aldrich, coordinator; *Mayo Clinic*: R. D. Brown, principal investigator, I. Meissner, co-principal investigator; D. Weibers, co-principal investigator; L. Jaeger, coordinator; *University of Michigan*: L. Morgenstern, principal investigator, L. Lisabeth, co-principal investigator, A. Caveney, coordinator; *New Jersey Medical School*: A. I. Qureshi, principal investigator, P. Harris-Lane, coordinator; *Northwestern University*: H. Batjer, principal investigator, C. Concannon, coordinator, G. Joven, coordinator, K. Matijevich, coordinator; *University of Ottawa*: M. T. Richard, principal investigator, A. Hopper, coordinator; *University of Pittsburgh*: A. B, Kassam, principal investigator, G. Seever, coordinator, J. Genevro, coordinator; *University of California*, *SF*: C. Johnston, principal investigator, K. Katsura, coordinator; *University of Southern California*: S. Giannotta, principal investigator, V. Thomson, coordinator, D. Fishback, coordinator; *Stanford University Medical Center*: G. Steinberg, principal investigator, D. Luu, coordinator; *University of Texas at Houston*: M. Malkoff, principal investigator, A. Wojner, coordinator; *University of Virginia*: N. Kassel, principal investigator, B. Worrall, co-principal investigator, S. Cook, coordinator B. Stoutenger, coordinator; *University of Washington*: D. Tirschwell, principal investigator, P. Tanzi, coordinator; *University of Manitoba (Winnipeg)*, A. Kaufmann, principal investigator, D. Gladish, coordinator; *Washington Universit:*C. Derdeyn, principal investigator, D. Rivet, co-principal investigator, M. Catanzare, coordinator, D. Gherardini, coordinator.

## Pre-publication history

The pre-publication history for this paper can be accessed here:


